# Foodborne Pathogens: Microbiology and Molecular Biology

**DOI:** 10.3201/eid1212.061077

**Published:** 2006-12

**Authors:** Sharon Balter

**Affiliations:** * New York City Department of Health and Mental Hygiene, New York, New York, USA

**Keywords:** Book review, foodborne pathogens, molecular biology, microbiology, Pina M. Fratamico, Arun K. Bhunia, James L. Smith

Foodborne pathogens can create a considerable amount of work at state and local health departments. Between foodborne outbreaks, restaurant inspections, environmental testing, botulism reports, customer complaints, and confirmation of isolates referred for testing, many health department resources are directed toward these pathogens and preventing illness from them. Moreover, the mass media are increasingly interested in food safety, particularly after large, multistate outbreaks caused by *Escherichia coli* O157:H7 and *Salmonella*, among other pathogens, and increasing public interest in raw and unpasteurized foods that are perceived as more natural or healthy. The audience for Foodborne Pathogens: Microbiology and Molecular Biology appears to be public health practitioners working on epidemiologic, environmental, and laboratory aspects of foodborne illness.

One of the book's strengths is that it attempts to include reference material on epidemiology and on the molecular and microbiologic aspects of the various pathogens. However, as the title suggests, the emphasis is on molecular and microbiologic aspects, and much of the information is extremely technical and primarily for the laboratory scientist. The book includes a range of food pathogens, from bacteria and viruses to mycotoxins. The primary omission is bovine spongiform encephalopathy. Chronic wasting disease is included briefly in a chapter on potential food pathogens, which makes the omission of bovine spongiform encephalopathy all the more striking.

In addition to separate chapters on individual pathogens or groups of pathogens, the book covers laboratory issues, including animal and cell culture models, molecular approaches for detection, and stress responses of foodborne pathogens. Other chapters are based on more sensational topics, such as bioterrorism and food, although this chapter discusses the subject in general terms. In a chapter on biosensor-based detection of foodborne pathogens, the authors conclude, not convincingly, that biosensors will soon be as widespread as glucose kits and home pregnancy tests.

Overall, the book is a good reference for health departments, especially the chapters on individual pathogens. However, the book could have used stronger editorial oversight. Books like this one, in which experts in highly specialized fields are each invited to write a chapter, will by their very nature lack an overriding point-of-view, but at the very least, the book should have had a strong introduction to put the content in context.

A large number of pathogens have emerged or been identified in the past 30 years, and a great deal of media attention is given to food-related illness. This book appears to be aimed at industrialized countries, despite the perception that the food supply in these countries is safe. Because much food is imported and exported throughout the world, including to and from industrialized nations, some basic discussion of the extent of foodborne illness in different parts of the world, and the resulting risk to the overall food supply, would have helped to frame the need for the book and the resources many health departments are putting toward foodborne illness.

